# Recent Developments in Upscalable Printing Techniques for Perovskite Solar Cells

**DOI:** 10.1002/advs.202200308

**Published:** 2022-03-10

**Authors:** Bhaskar Parida, Arjun Singh, Abdul Kareem Kalathil Soopy, Sambasivam Sangaraju, Madhulita Sundaray, Satrujit Mishra, Shengzhong (Frank) Liu, Adel Najar

**Affiliations:** ^1^ Department of Physics College of Science United Arab Emirates University Al Ain 15551 UAE; ^2^ Department of Applied Sciences The Northcap University Gurugram 122017 India; ^3^ Department of Humanities and Sciences KG Reddy College of Engineering and Technology Hyderabad 501504 India; ^4^ Department of Physics Parala Maharaja Engineering College Berhampur Odisha 761003 India; ^5^ Dalian National Laboratory for Clean Energy iChEM Dalian Institute of Chemical Physics Chinese Academy of Sciences Dalian Liaoning 116023 China; ^6^ Key Laboratory of Applied Surface and Colloid Chemistry Ministry of Education Shaanxi Engineering Lab for Advanced Energy Technology School of Materials Science and Engineering Shaanxi Normal University Xi'an Shaanxi 710119 China

**Keywords:** blade, coating, perovskite, printing, solar cell

## Abstract

Just over a decade, perovskite solar cells (PSCs) have been emerged as a next‐generation photovoltaic technology due to their skyrocketing power conversion efficiency (PCE), low cost, and easy manufacturing techniques compared to Si solar cells. Several methods and procedures have been developed to fabricate high‐quality perovskite films to improve the scalability and commercialize PSCs. Recently, several printing technologies such as blade‐coating, slot‐die coating, spray coating, flexographic printing, gravure printing, screen printing, and inkjet printing have been found to be very effective in controlling film formation and improving the PCE of over 21%. This review summarizes the intensive research efforts given for these printing techniques to scale up the perovskite films as well as the hole transport layer (HTL), the electron transport layer (ETL), and electrodes for PSCs. In the end, this review presents a description of the future research scope to overcome the challenges being faced in the printing techniques for the commercialization of PSCs.

## Graphical abstract

In the last few years, impressive efforts have been made on scalable printing techniques for the fabrication of highly efficient perovskite solar cells (PSCs) and their commercialization. This review highlights on the scalable growth of high‐quality perovskite films, carrier transport layers, and electrodes using several printing techniques. Also, forthcoming challenges, and scopes of these printing techniques are offered.

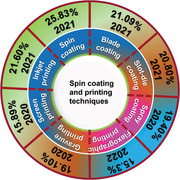

## 1 Introduction

The global energy requirements are increasing rapidly, which is mostly full filled with exhaustible nonrenewable fossil fuels. However, the generation of electrical energy from these fossil fuels has detrimental effects on human health and the environment. Therefore, renewable energy such as wind energy, water energy, wave energy, geothermal energy, hydrogen biomass, and solar energy have been found to be the best sources to produce electrical energy. Among these renewable energy sources, solar energy is the most abundant energy on earth, which can play a pivotal role in achieving carbon neutrality and reducing global warming in the coming years. Therefore, in the last few decades, the research and development of solar cells have been increased, which significantly dropped the solar cell price to a few cents.^[^ ^1^ ^]^ It is crucial to further accelerate the global installation of PV modules and reduce the levelized costs of PV energy (LCOE). However, the current photovoltaic market is dominated by crystalline silicon (Si) based solar cells due to their mature technology, low cost, and superior stability over 25 years, and high PCE up to 26.7%.^[^ ^2^ ^]^ The monocrystalline and the polycrystalline Si solar cells are presently being commercialized and have a full‐size large area power conversion efficiency (PCE) of more than 20%.^[^ ^3^ ^]^ However, Si solar cells are still expensive despite their well‐established technology and long‐term stability.^[^ ^4^ ^]^ Therefore, various studies have been made to develop low‐cost thin‐film and flexible solar cells using the solution and low‐temperature processes.

Just over a decade, metal halide perovskites have been emerged as promising materials for solar cell application due to their low exciton binding energy,^[^ ^5^, ^6^ ^]^ fast carrier diffusion speed,^[^ ^7^, ^8^ ^]^ long diffusion length,^[^ ^9^, ^10^ ^]^ high absorption coefficient,^[^ ^11^, ^12^ ^]^ and wide absorption window.^[^ ^13^, ^14^ ^]^ In 2009, Kojima et al. first introduced MAPbBr_3_ perovskite as a light sensitizer for dye‐sensitized solar cells and achieved a PCE of 3.8%.^[^ ^15^ ^]^ However, the stability of these solar cells was very low (in a matter of seconds) due to the use of liquid electrolytes, which could dissolve the perovskite under the light. In 2012, Kim et al. introduced 2,2^′^,7,7^′^‐tetrakis[N,N‐di(4‐methoxyphenyl)amino]‐9,9^′^‐spirobifluorene (spiro‐OMeTAD) as hole transporting layer (HTL) which significantly enhanced the PCE of the PSCs to around 10% with stability over 500 h.^[^ ^16^, ^17^ ^]^ Thereafter, full‐fledged research activity on PSCs has been developed across the globe. In 2014, PSCs became a new category of emerging photovoltaic technology in the National Renewable Energy Laboratory (NREL)’s Best Solar Cells Efficiency chart with a record PCE of 14.1%.^[^ ^18^ ^]^ Consequently, the PCE of the PSCs gradually increased through several strategies.^[^ ^19^ ^]^ Thus, the PCE of PSCs tremendously increased from 3.8% to over 25.5% recently in just over a decade.^[^ ^20^, ^21^ ^]^ Similarly, tandem devices combining Si and PSCs have exceeded the Shockley‐Queisser limit and achieved a PCE of over 29%.^[^ ^22^ ^]^

These record‐breaking PCEs have been achieved on a lab scale with small‐area devices (<1 cm^2^). However, devices with large‐area (>100 cm^2^) are required for the commercialization of PSCs.^[^ ^23^ ^]^ Therefore, to upscale and commercialize PSCs, other fabrication techniques such as D bar blade coating,^[^ ^24^ ^]^ slot die coating,^[^ ^25^ ^]^ spray coating,^[^ ^26^ ^]^ solvent‐free stamping,^[^ ^27^ ^]^ vacuum‐deposition methods,^[^ ^28^ ^]^ and various printing methods were utilized by several researchers.^[^ ^13^, ^29^ ^]^ Especially, printing methods are more effective in fabricating large‐area components with high throughputs at low cost and can be highly beneficial to manufacturing large‐scale PSCs. As attention has been driven to improve the PCE of the PSCs from laboratory scale to commercial scale, a maximum PCE over 21% has been achieved by printing technology to date.^[^ ^30^ ^]^ However, less attention has been focused on the printing technologies relative to spin coating; even these printing techniques are more likely to be adopted for the scalability and commercial production of PSCs.

This review highlights the recent advances in printing technologies adopted for the fabrication and commercialization of large‐size PSCs. In particular, we discuss various printing techniques that have been utilized to fabricate highly efficient PSCs by controlling the quality of perovskite absorber, HTL, ETL, and electrode. We also discuss the device architecture for developing efficient PSCs. In the end, a summary of the current challenges and future directions for improving the PCE and stability of the large‐area printed PSCs will be presented.

## 2 Device Architecture of Perovskite Solar Cell

**Figure 1:**
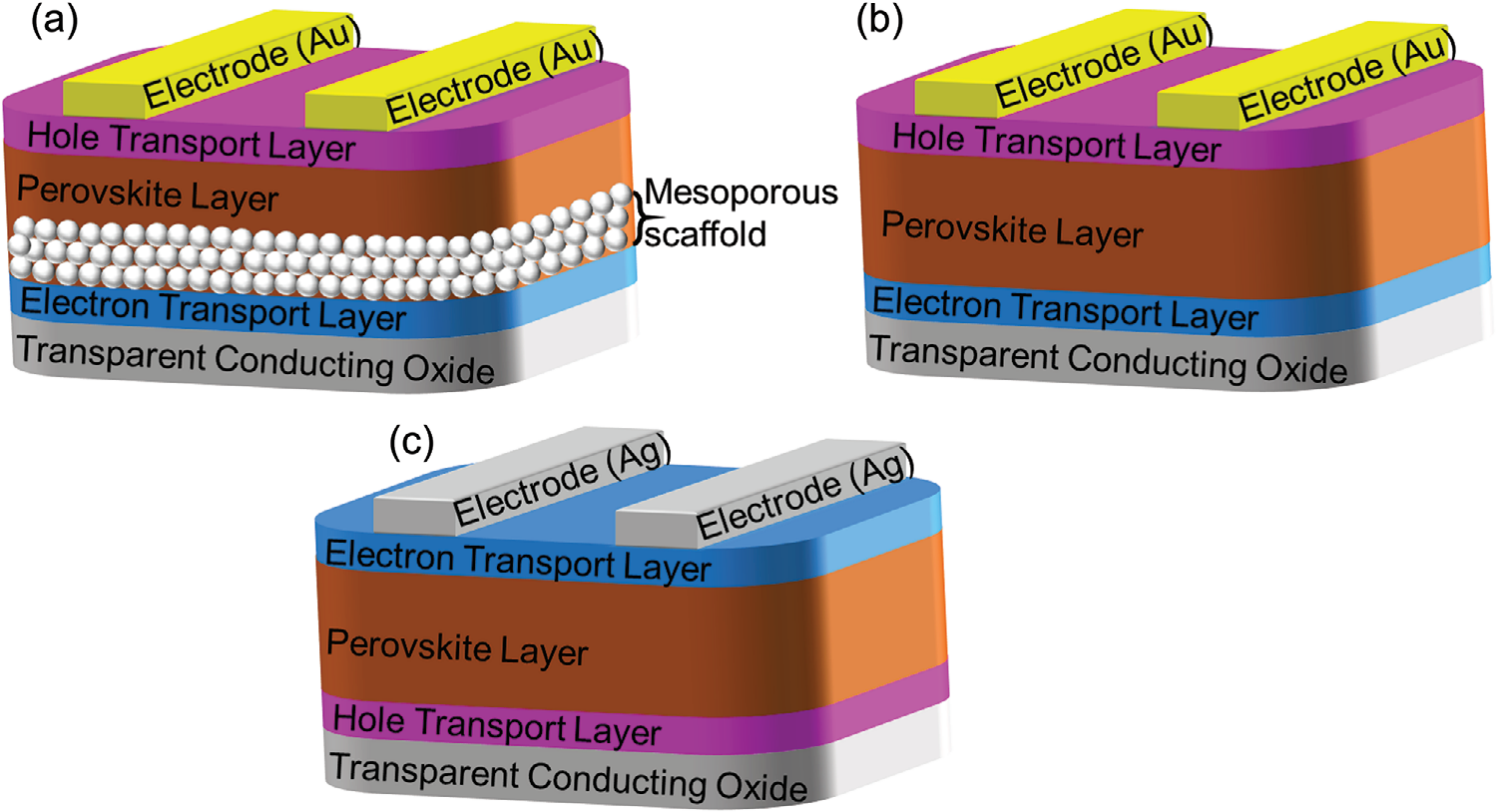
Schematic architecture of a) mesoporous, b) regular planar, and c) inverted planar PSCs.

The typical formula of the perovskite is depicted as ABX_3_, where X is a halide (I^−^, Br^−^, and Cl^−^), A is the monovalent cation (Cs^+^, MA^+^, FA^+^, Rb^+^), and B site is a divalent cation (Pb^2+^, Sn^2+^_,_ Cu^2+^, Mn^2+^).^[^ ^31^ ^]^ Also, the crystal structure and the stability of the perovskites can be determined from the tolerance factor *t* and octahedral factor *μ*, where *t* is characterized as the ratio of the distance A‐X to the distance B‐X in an idealized solid‐sphere model $t\;=\;\frac{R_{A}+\;R_{X}}{\surd 2(R_{B}+R_{X})}$, and *μ*  =  *R*_B_/*R*_X_ where *R*_A_, *R*_B,_ and *R*_X_ are the radius of the A‐site, B‐site cation, and X‐site anion, respectively.^[^ ^32^ ^]^ When 0.8 ≤ *t* ≤ 1 and 0.44 < *μ* < 0.9, a perfect and stable black phase of the perovskite can be obtained, whereas an ideal cubic symmetry of perovskite requires a *t*‐factor between 0.9 and 1. Thus, lattice distortion can be occurred by lowering or exceeding the upper limit of the *t*‐factor.^[^ ^33^ ^]^ The lattice distortion owes a non‐perovskite yellow phase which exhibits worse optical absorption wider bandgap, therefore, not suitable for optoelectronic devices. Moreover, the PSC consists of multiple layers: a transparent conducting oxide as a front electrode, the perovskite absorber sandwiched between an ETL and HTL, and a metal back electrode. Three different structures, such as mesoporous and the planar heterostructure of PSCs (**Figure** **1**) will be discussed.

### 2.1 Mesoporous Structure

The light‐receiving area of the photosensitive material needs to be increased to improve the device efficiency. As mesoporous material exhibits a large specific surface area of up to 1000 m^2^ g^‐1^ and high porosity, these are considered the best for device efficiency.^[^ ^34^ ^]^ The mesoporous layer allows the infiltration of perovskite precursors to form an adequate thickness of the perovskite active layer. Several mesoporous materials, including TiO_2_, Al_2_O_3,_ and ZrO_2,_ are highly used for the mesoporous PSCs.^[^ ^35^, ^36^, ^37^ ^]^ However, the PSC with the mesoscopic structure containing mesoporous TiO_2_ as an ETL has delivered a maximum efficiency of 25.6%.^[^ ^21^ ^]^ The mesoporous PSCs generally consists of a dense TiO_2_ compact layer deposited on the transparent conducting oxide (TCO) surface (fluorine‐doped tin oxide, FTO), a mesoporous TiO_2_ layer, which is thoroughly infiltrated by the perovskite absorber, a perovskite overlayer, an organic hole transport layer and a metal electrode (Figure 1a). The high‐temperature sintering process of the mesoporous PSCs hampers the development of low‐cost, flexible, and perovskite‐based tandem devices. Also, TiO_2_ can easily provoke the degradation of the perovskite structure under exposure to UV illumination as another known challenge for the commercialization of PSCs. Thus, several research efforts have been made to fabricate low‐temperature processed mesoporous PSCs and planar PSCs.^[^ ^38^, ^39^, ^40^, ^41^ ^]^ In addition, some other strategies such as interfacial engineering^[^ ^42^, ^43^, ^44^ ^]^ and metal doping^[^ ^45^, ^46^, ^47^, ^48^, ^49^ ^]^ have been employed to increase the PCE of the mesoporous PSCs. Similarly, for the conventional n–i–p structured mesoporous PSCs, SnO_2_ scaffolds have been utilized as ETLs, which delivered a PCE of over 22%.^[^ ^50^, ^51^ ^]^ Also, inverted p–i–n structured mesoporous PSCs have been reported to employ mesoporous NiO*_x_* and CuGaO_2_ HTLs that effectively extract holes from perovskite due to the increased contact area of the perovskite/HTL, resulting PCE approximately 20%.^[^ ^52^, ^53^ ^]^

### 2.2 Planar Heterostructure

Initially, the metal halide perovskite exhibited a short charge diffusion length (<20 nm). Thus, the mp‐TiO_2_ was used as ETL to facilitate carrier transportation from the perovskite efficiently. However, in later findings, it has been reported that the perovskite exhibits a diffusion length of over 100 nm,^[^ ^10^ ^]^ which leads to the possibility of the planar PSCs. The absence of porous metal oxide in planar heterostructures shows significantly different results from the mesoporous structure. In planar heterostructures, two different interfaces at the active perovskite/ETL and perovskite/HTL are formed. The planar heterostructure (Figure 1b) of the perovskite solar cells can increase the light absorption, which facilitates an increase in the efficiency and flexibility of the device. The planar heterojunction PSCs with compact TiO_2_ as ETL was first reported by Snaith et al. in 2012 with a PCE of 1.8%.^[^ ^17^ ^]^ They further analyzed that the poor performance of the planar structure was mainly due to the poor coverage of perovskite crystals. Later, they optimized the solution‐based film formation and achieved a PCE of 11.4% using only compact TiO_2_ ETL.^[^ ^54^ ^]^ For the planar PSCs, TiO_2_ and SnO_2_ are the most commonly used ETLs because of their favorable band alignment and low‐temperature processing, although several other materials such as ZnO,^[^ ^55^ ^]^ Nb_2_O_5_,^[^ ^56^ ^]^ WO_3_,^[^ ^57^ ^]^ MoS_2_,^[^ ^58^ ^]^ PCBM,^[^ ^59^ ^]^ and C_60_^[^ ^60^ ^]^ are used as ETLs.^[^ ^61^, ^62^ ^]^ Recently, Yoo et al. tuned the chemical bath deposition of SnO_2_ based ETLs for the planar PSCs and achieved a PCE of over 24%. The same group further reported the passivation of the bulk and interface of the perovskite (FAPbI_3_) layer with small amounts of MAPbBr_3_ and 2‐D perovskite, respectively, which improved the PCE to over 25.2%,^[^ ^63^ ^]^ Recently, Min et al. reported the passivation of SnO_2_ and perovskite interface using the Cl‐containing perovskite precursor which atomically passivated the interfacial defects and improved charge extraction and transportation from the perovskite layer. As a result, they achieved a PCE of 25.83%, which is the highest PCE of PSCs reported to date.^[^ ^20^ ^]^ Recently, Kim et al. reported the replacement of mesoporous TiO_2_ ETL with a thin layer of polyacrylic acid‐stabilized tin(IV) oxide quantum dots (paa‐QD‐SnO_2_) on the compact‐TiO_2,_ which significantly enhanced the light capture and suppressed the nonradiative recombination at the ETL/perovskite interface. As a result, they achieved a PCE of 25.7% (area of 0.08 cm^2^) with high operational stability. Also, the use of paa‐QD‐SnO_2_ enabled the fabrication of highly efficient PSCs with larger area of 1, 20, and 64 cm^2^ which delivered PCEs of 23.3, 21.7, and 20.6%, respectively.^[^ ^64^ ^]^

Similarly, the first inverted planar heterojunction PSCs (Figure 1c) with PCE of 3.9% was reported by Guo et al. in 2013.^[^ ^65^ ^]^ Later, Malinkiewicz et al. introduced poly(3,4‐ethylene dioxythiophene): polystyrene sulfonate (PEDOT: PSS) as the HTLs and prepared low‐temperature processed planar inverted PSCs to obtain a PCE of 12%.^[^ ^66^ ^]^ This low‐temperature process led to fabricating inverted planar PSCs on flexible substrates. Also, some other organic HTL materials, including Spiro‐OMeTAD,^[^ ^35^ ^]^ PTAA,^[^ ^67^ ^]^ P3HT,^[^ ^68^ ^]^ and inorganic HTLs such as NiO*_x_*,^[^ ^69^ ^]^ MoO*_x_*,^[^ ^70^ ^]^ VO*_x_*,^[^ ^71^ ^]^ CuSCN,^[^ ^72^ ^]^ CuI,^[^ ^73^ ^]^ Cu_2_O, and CuO^[^ ^74^ ^]^ have been developed for the inverted planar PSCs. However, the interfacial diffusion of the organic molecules and chemical/thermal stability issues opened a new window to explore the inorganic HTL materials. Among various inorganic HTL materials, NiO*_x_* is efficiently used because of its excellent properties such as optical transmittance, chemical stability, wide bandgap, high mobility, favorable matching energy band alignment with perovskites, and low cost. The first NiO*_x_* HTL based inverted planar PSCs resulted in a PCE of less than 10%, which was lower than its organic counterparts due to its poor conductivity.^[^ ^75^ ^]^ Therefore, Cu,^[^ ^76^ ^]^ Cs,^[^ ^77^ ^]^ Co,^[^ ^78^ ^]^ Li,^[^ ^79^ ^]^ Mg,^[^ ^80^ ^]^ and Al^[^ ^59^ ^]^ metals were doped, which increased the hole conductivity and work function. As a result, the best efficiency of over 20% has been achieved.^[^ ^59^ ^]^ In addition, the dilemma between efficient out‐of‐plane electron transition and the in‐plane full film coverage of the fullerene‐based top buffer layers also limits the substantial development of inverted planar PSCs.^[^ ^81^ ^]^ Recently, Hu et al. developed 0D:1D composites using C_60_ and M‐TiO_2_ (M = Fe, Co) for the top buffer layer of the inverted planar PSCs that improved the film uniformity, electron extraction, and transfer ability, energy level matching with perovskite, and the stability of the devices. Furthermore, this group also reported the highest PCE exceeding 22% with a 22‐fold prolonged working lifetime.^[^ ^82^ ^]^ The above‐mentioned device structures have been fabricated by the conventional spin‐coating technique. Consequently, several printing techniques have also been used to fabricate small and large‐scale PSCs. For example, the screen printing technique is widely used for the fabrication of mesoporous PSCs,^[^ ^15^, ^83^, ^84^, ^85^ ^]^ while gravure printing and inkjet printing techniques are used for the fabrication of planar PSCs,^[^ ^86^, ^87^, ^88^, ^89^, ^90^ ^]^ which will be discussed in the next section.

## 3 Printing Techniques for PSCs

To date, the highly efficient PSCs have been made by using small substrates with a spin‐coating process. The spin coating process involves the spreading of perovskite precursors on the substrate via shear force. However, the reproducibility of spin‐coated PSCs varies between research laboratories. In addition, the spin‐coating of the perovskite precursor on the large substrates results in nonuniform thickness due to the waste of solution during coating, which hampers the device performance. Therefore, spin‐coating is not unsuitable for the mass production and commercialization of PSCs. Several efforts have been made to develop different coating technologies for the scalability and mass production of PSCs.^[^ ^26^, ^29^, ^91^ ^]^

**Figure 2:**
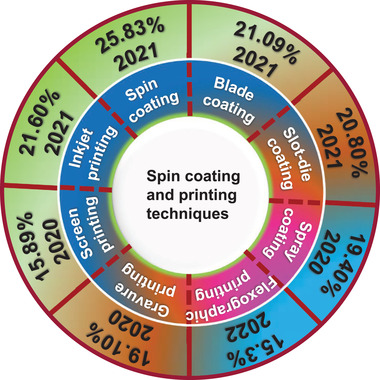
An overview of the highest PCE of PSCs using spin‐coating and different printing techniques.

Although these technologies have increased the performance of large‐area PSCs, there are still many problems like antisolvent extraction in a one‐step method, the utilization rate of materials, and suitability for roll‐to‐roll processing.^[^ ^92^, ^93^ ^]^ To overcome these issues for scalability of PSCs and commercialization of PSCs, different printing technologies (shown in **Figure** **2**) such as blade‐coating, slot‐die coating, spray‐coating, flexographic printing, gravure printing, screen‐printing, and inkjet printing have been developed. Among these printing techniques, inkjet printing delivered a maximum PCE of 21.60%. The recent developments in these printing techniques for upscaling the PSCs will be discussed in this section.

### 3.1 Blade Coating

**Figure 3:**
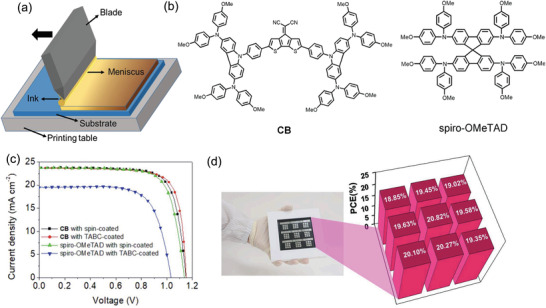
a) Schematic illustration of blade coating technique. b) Chemical structure of CB and spiro‐OMeTAD. c) *J*–*V* curve of the PSCs (FTO glass/c‐TiO_2_/mp‐TiO_2_/CH_3_NH_3_PbI_3_/CB/Ag) prepared with spray‐coating technique and d) photographic image of 10 cm × 10 cm large‐area devices fabricated by TABC and the schematic illustration of the PCE distribution on it. Reproduced with permission.^[^ ^122^ ^]^ Copyright 2021, Elsevier.

Blade coating, also known as knife coating or bar coating, is a powerful printing technique using low‐cost equipment to achieve large area coating. As it is suitable for both rigid and flexible substrates, it has become one of the most popular printing techniques for thin‐film coatings. To deposit a thin film, the ink is directly loaded onto the substrate, and a knife‐type blade coater is used to spread the ink on the substrate. For this type of coating, either the blade or the substrate moves to create a homogenous wet thin film (**Figure** **3**a). Therefore, the thickness of the deposited thin film is mainly determined by the meniscus formed by the solution in between the blade and the substrate, as well as the concentration of the ink. The meniscus can be controlled by the gap between the blade and the substrate, the speed of the blade relative to the substrate, the viscosity of the ink, the geometry of the blade, and the substrate wettability.^[^ ^94^ ^]^ Similarly, the blade may be designed with different shapes, like a cylindrical bar known as D‐bar coating.^[^ ^95^ ^]^

Several groups have extensively reported blade coating as a single‐step deposition technique for PSCs.^[^ ^96^, ^97^, ^98^, ^99^, ^100^, ^101^, ^102^, ^103^, ^104^, ^105^, ^106^, ^107^, ^108^, ^109^, ^110^, ^111^, ^112^ ^]^ However, controlling the crystallization of the perovskite film during the blade coating process is more challenging than the spin‐coating process as the evaporation of the solvent is relatively slow. Therefore, it is quite difficult to form a dense uniform solid film by the blade‐coating method as the ink chemistry may change during the processing time, which is not suitable for improving the PCE of the PSCs.^[^ ^99^ ^]^ To overcome the solvent drying issue, He et al. reported the meniscus‐assisted blade‐coating method where a concave meniscus was formed between the substrate and the blade.^[^ ^98^ ^]^ This method suggests that the fast solvent evaporation at the edge of the meniscus facilitated an outward convective flow to refill the loss of solvent by evaporation and promoted the nucleation and growth of micrometer‐sized perovskite crystals by transporting the perovskite solutes to the contact line. Thus, the perovskite film produced by the meniscus‐assisted blade coating method exhibits excellent optoelectronic properties with an efficiency of 19.23%. In addition, surfactant‐controlled ink with substrate preheating has been found to be an effective approach to scale up the solution‐based deposition of perovskite films by the blade‐coating method. The pre‐heating of the substrate was found to be beneficial for faster evaporation of the solvent and to suppress the formation of a needle‐like structure. In contrast, the surfactant (L‐*α*‐phosphatidylcholine) dramatically altered the fluid drying dynamics and increased the adhesion of perovskite ink to the underlying charge transport layer. The additive also enabled the blade coating of perovskite film at a speed of 180 m h^–1^ and passivated the carrier traps, resulting in an efficiency over 20% with small area devices, 15.3% and 14.6% for modules with aperture areas of 33.0 and 57.2 cm^2^, respectively.^[^ ^92^ ^]^ Similarly, another strategy of rapid drying of solvent was reported by creating a low boiling point solvent system such as acetonitrile and methyl acetate.^[^ ^110^ ^]^ Moreover, a mixture of 2% volatile‐non‐coordinated solvent system and nonvolatile coordinated solvents enhanced the grain size due to the faster drying of the solvent during coating, which resulted in a certified efficiency of 16.4% with an area of 63.7 cm^2^.^[^ ^109^ ^]^ Apart from these physical and chemical modification approaches, several other approaches such as solvent engineering, adding, and doping strategies have also been summarized well in the previous reports.^[^ ^24^, ^113^ ^]^ Furthermore, several groups have successfully developed blade‐coating technique for printing of not only HTLs like the polymers PEDOT:PSS,^[^ ^112^, ^114^, ^115^, ^116^ ^]^ and PTAA^[^ ^117^, ^118^ ^]^ or small molecules like Spiro‐OMeTAD^[^ ^119^, ^120^, ^121^, ^122^ ^]^ and NiO*_x_*,^[^ ^123^, ^124^, ^125^ ^]^ but also electron transport layers (ETLs) like the fullerenes C_60_, ^[^ ^112^ ^]^ PCBM,^[^ ^112^, ^124^, ^126^, ^127^ ^]^ ZnO,^[^ ^128^ ^]^ SnO_2_,^[^ ^129^, ^130^, ^131^, ^132^ ^]^ and TiO_2_. ^[^ ^133^ ^]^ Recently, Lee et al. developed a new donor–acceptor–donor type HTL with 4‐dicyanomethylene‐4H‐cyclopenta[2,1‐b;3,4‐b’]dithiophene (diCN‐CPDT) core tethered with two bis(alkoxy) diphenylaminocarbazole periphery groups (Figure 3b) and applied it for the fabrication of fully printed perovskite solar cell using a thermal assisted blade‐coating technique.^[^ ^122^ ^]^ These fully printed PSCs delivered a PCE as high as 21.09% (Figure 3c), the highest PCE obtained from fully printed PSCs without using dopant in the HTL. Meanwhile, they also obtained an average PCE of 19.68% for the devices fabricated on a 10 cm × 10 cm substrate, and the devices (Figure 3d) showed excellent long‐term stability of more than 94% after 500 h of storage in ambient conditions with ≈50% relative humidity without encapsulation.

It has been reported that during blade‐coating, the perovskite tends to form intermediate solvates with solvents at low temperatures, which leads to poor surfaces and the formation of traps. Li et al. developed a Phase Transition Control for high‐performance blade‐coated perovskite solar cells, which effectively suppressed the intermediate formation with a direct phase transformation from the sol‐gel to the perovskite nucleus and crystals.^[^ ^96^ ^]^ Similarly, Chang et al. developed a series of green solvents for ecofriendly printing perovskite solar cells via blade‐coating under ambient conditions.^[^ ^104^ ^]^ The microstructure and lattice strain control enabled the efficiency to improve from 18.26% to 20.21%, comparable to the efficiencies of the state‐of‐the‐art MAPbI_3_‐based cells fabricated from toxic solvents.^[^ ^134^ ^]^ Using the blade‐coating process, Fan et al. developed a scalable ambient fabrication of high‐performance CsPbI_2_Br solar cells with an efficiency of 14.7% for small‐aperture‐area (0.03 cm^2^) and 12.5% for the large‐aperture‐area (1.0 cm^2^) ones, the highest at the time for large‐area all‐inorganic perovskite solar cells.^[^ ^102^ ^]^ It is known that the conflict between air‐flow‐assisted fast drying and low‐quality film causes energy misalignment and trap formation. Chang et al. developed an additive strategy to deliver a PCE of 19%, the highest record at the time for all CsPbI_3_ solar cells.^[^ ^103^ ^]^ By incorporating a low concentration of novel ionic liquid, the CsPbI_3_ lattice strain generated during film formation was efficiently decreased. The PCE of ambient printed CsPbI_3_ solar cells was further improved to 20.01%.^[^ ^135^ ^]^

### 3.2 Slot‐Die Coating

**Figure 4:**
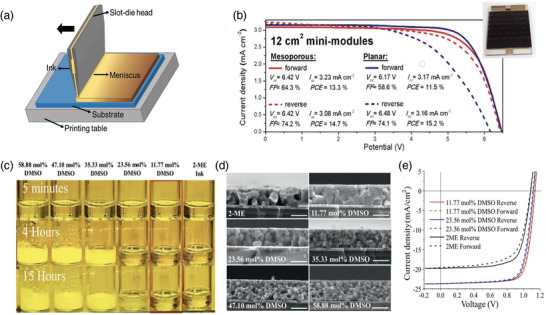
a) Schematic illustration of slot‐die coating technique. b) *J*–*V* curves of the champion modules with a device area of 12 cm^2^ for both mesoporous (FTO glass/c‐TiO_2_/m‐TiO_2_) and planar (FTO glass/c‐TiO_2_/SnO_2_) architecture. Reproduced with permission.^[^ ^146^ ^]^ Copyright 2021, Wiley‐VCH. c) Photographic images of 2‐ME DMSO solvents‐based perovskite inks (in store inside the glovebox). d) SEM cross‐section images of the slot‐die coated perovskite films prepared using 2‐ME DMSO solvents‐based perovskite inks and e) *J*–*V* curves of the PSCs (ITO glass/SAM/MAPbI_3_/C_60_/BCP/Cu) fabricated by the slot‐die coating of using 2‐ME DMSO solvents‐based perovskite inks. Reproduced under the Creative Common CC By license.^[^ ^150^ ^]^ Copyright 2021, The Authors, published by Wiley‐VCH.

Slot‐die coating is one of the best printing technologies to increase the automation and reproducibility of the printing with a patterned layout by controlling the amount of ink. Slot‐die coating is a process particularly suitable for roll‐to‐roll processes as the ink is continuously supplied through a thin slit to spread over the moving substrate (**Figure** **4**a). The slot‐die coater consists of an ink reservoir that continuously supplies ink to two independently moveable metal blades forming a slit on the bottom side which is placed with a fixed gap over the substrate. The ink reservoir is usually connected to a solution pumping system that supplies ink at a controlled rate. During deposition, an upstream and downstream meniscus of the solution is formed between the head lips and the substrate. While coating, the ink is pumped into the head and filling the slit; the solution contacts the substrate and forms a meniscus between the substrate and the coating head to print a continuous film. Compared to blade‐coating, the slot‐die coating requires more ink to fill up the reservoir. However, the mechanism of slot‐die coating is quite similar to the blade coating. Several models such as the capillary model, viscous model, and viscous capillary model have been proposed to explain the operational process that affects the slot‐die coating for many pioneering works.^[^ ^136^ ^]^ The operating limit like low‐flow limit, over‐flow limit, and air‐entrainment defects are well summarized in the previous report.^[^ ^25^ ^]^ Notably, it is essential to control the operating parameters such as the gap between the slot‐die head, the surface tension, the flow rate, and the concentration of the ink to print a film with uniform thickness using the slot‐die coating.^[^ ^137^ ^]^

Similar to the blade‐coating process, both one‐step and two‐step deposition methods are feasible for the sheet‐to‐sheet and roll‐to‐roll deposition of the perovskite layer by the slot‐die coating process.^[^ ^138^, ^139^, ^140^, ^141^, ^142^, ^143^, ^144^, ^145^ ^]^ For example, in 2015, Hwang et al. first reported a sequential slot‐die coating process to fabricate fully printed PSCs with device configuration of ITO/ZnO/MAPbI_3_/P3HT/Au.^[^ ^144^ ^]^ For the MAPbI_3_ perovskite film, they sequentially slot‐die coated PbI_2_ film and then an MAI layer on the top. By optimizing the process temperature of MAI coating, they achieved a PCE of 12%, which was comparable to that of spin‐coated devices. Recently, Zimmermann et al. utilized slot‐die coating for the sequential deposition of PbI_2_ mixed with CsI, followed by coating the organic cations such as FAI, MAI, MABr, and MACl, on the top for the preparation of triple cation based perovskite (Cs_0.05_MA_0.4_FA_0.55_Pb(I_0.96_Br_0.04_)_3_) on a large substrate with an area of 5 × 10 cm^2^.^[^ ^146^ ^]^ By optimizing the ink composition and deposition parameters they achieved a PCE of 19% for small PSCs (area < 1cm^2^) with planar device structure of FTO/TiO_2_/SnO_2_/Cs_0.05_MA_0.4_FA_0.55_Pb(I_0.96_Br_0.04_)_3_/spiro‐OMeTAD/Au and 15.2% for minimodules with device area of 12 cm^2^ (Figure 4b). Similarly, the substrate temperature and the gas‐blowing during the coating process significantly influence the morphology and the PCE of the PSCs.^[^ ^141^, ^147^, ^148^ ^]^ For example, Zuo et al. reported the slot‐die coating of NH_4_Cl added MAPbI_3_ perovskite films on both glass and flexible PET substrates with the combinational approach of substrate preheating (60 °C) and nitrogen gas blowing. Interestingly, the devices with the configuration of ITO/PEDOT:PSS/MAPbI_3_/PCBM/Ag delivered PCEs of 15.6% for glass PSCs and 11.2% for roll‐to‐roll flexible PSCs.^[^ ^147^ ^]^ Using the same substrate heating and gas quenching method, Fievez et al. reported the slot‐die coating of methyl ammonium‐free Cs_0.16_FA_0.84_Pb(I_0.88_Br_0.12_)_3_ perovskite film for the fabrication of n–i–p structured PSCs which delivered a PCE of 18% over 0.09 cm^2^ active‐area device.^[^ ^149^ ^]^ Moreover, optimization of printing process technology and ink formulation play a pivotal role in scaling up PSCs with reproducibility. Therefore, Li et al. prepared MAPbI_3_ perovskite ink using 2‐methoxy‐ethanol and DMSO solvents and slot‐die coated to fabricate PSCs. Optimizing the ink formulation (by adding 11.77 mol% of DMSO) and aging time (Figure 4c,d), they achieved a PCE of 20.8% and 15% for device area of 0.16 cm^2^ and 2.2 cm^2^, respectively (Figure 4e).^[^ ^150^ ^]^ Also, the slot‐die coating of not only the perovskite layer but also the HTL, ETL, and electrodes can be beneficial for high‐throughput mass production, upscaling, and commercialization of the PSCs. In this regard, organic and inorganic HTLs like Spiro‐OMeTAD, Bifluo‐OMeTAD,^[^ ^151^ ^]^ and NiO*_x_*,^[^ ^152^ ^]^ as well as ETLs such as TiO_2_,^[^ ^138^, ^153^ ^]^ SnO_2_^[^ ^154^ ^]^ and carbon electrode^[^ ^153^ ^]^ have been successfully slot‐die coated for upscaling the PSCs. These results indicate the potential of slot‐die coating for scaling up PSCs. Recently, Du et al. reported their success using a slot‐die coating to achieve solar cell efficiency as high as 22.7%, and a 40 × 40 mm^2^ module attained stabilized PCE as high as 19.4%, both are among the highest made using large‐area fabrication process.^[^ ^155^ ^]^

### 3.3 Spray Coating

**Figure 5:**
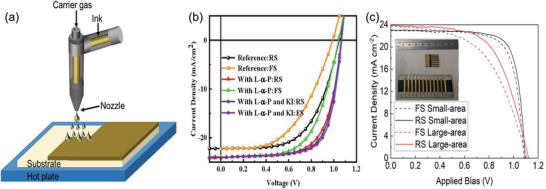
a) Schematic illustration of the spray‐coating technique. b) *J*–*V* curves of the spray‐coated PSCs (ITO glass/SnO_2_/perovskite/spiro‐OMeTAD/Ag) with L‐*α*‐phosphatidylcholine and KI additives for the perovskite ink. Reproduced with permission. ^[^ ^159^ ^]^ Copyright 2021, American Chemical Society. c) *J–V* curves of the fully spray‐coated PSCs (ITO glass/SnO_2_/perovskite/spiro‐OMeTAD/Ag). Reproduced under the Creative Common Attribution 4.0 International license.^[^ ^167^ ^]^ Copyright 2020, The Authors, published by Nature.

Spray coating is another versatile solution‐based low‐temperature coating technology that has been widely used to deposit high‐quality thin films over large area substrates. Spray coating consists of an ultrasonic nozzle that disperses the ink into tiny liquid droplets. These tiny droplets are directed as a spray onto the substrate using a compressed gas jet (**Figure** **5**a). These droplets adhere together through the coalescence process to form a uniform wet film. A solid film can be obtained as the solvent evaporates by the drying process.^[^ ^156^ ^]^ The size and uniformity of the droplets influence the uniformity of the film. The uniformity of both the droplets and the film can be affected by the properties of ink, the nozzle type, the flow rate of the ink in the nozzle, and the gas pressure. Therefore, ink with low surface tension and a small contact angle is crucial for improving surface wetting, making it the most complex process among all scalable techniques.

Spray coating has also been successfully implemented for the fabrication of PSCs. For example, in early 2014, Barrows et al. first introduced a spray‐coating method to fabricate PSCs. They studied the substrate temperature, ink properties, and post‐annealing process to improve the perovskite film quality and achieved a PCE of 11.1% for the small area devices (0.025 cm^2^).^[^ ^157^ ^]^ Since then, many developments have been made to fabricate PSCs using the spray‐coating process, which is well described in the previous report.^[^ ^26^ ^]^ Recently, Heo et al. developed the spray‐coating of graded CsPbI_3‐_ *_x_* Br*_x_* inorganic perovskite films and achieved PCEs of 16.81% for small devices (0.096 cm^2^) and 13.82% for minimodules with an area of 112 cm^2^. Also, the submodules showed excellent stability after 1000 h continuous light soaking.^[^ ^158^ ^]^ Similarly, Gao et al. reported the binary additive engineering strategy for spray‐coating (FAPbI_3_)*_x_* (MAPbBr_3_)_1–_ *_x_* hybrid perovskite in air. By successfully adding L‐*α*‐phosphatidylcholine and KI into the perovskite ink, they found that the L‐*α*‐phosphatidylcholine narrowed the grain boundary, and KI further reduced the grain boundaries and passivated the defects, which significantly improved the PCE (18.2%) (Figure 5b).^[^ ^159^ ^]^ It is very challenging to achieve high‐quality perovskite films with uniform morphology and homogeneous crystallinity due to the coffee ring effect in the spray‐coating process. To overcome this issue, a two‐step process combining evaporation and spray coating has been found to be very effective in depositing Cs_0.19_FA_0.81_PbI_2.5_Br_0.5_ perovskite films. The application of spray‐coating has been found to be feasible for the deposition of HTL, ETL, and electrodes. He et al. reported a scalable and high‐performance ZnO‐SnO_2_ cascade double‐layer ETL employing the spray‐coating process for an efficient and stable perovskite module. They achieved a PCE of 17.8% and 16.6% for the module sizes of 6 × 6 cm^2^ and 10 × 10 cm^2^, respectively.^[^ ^131^ ^]^ In addition, spray‐coating of SnO_2_,^[^ ^160^, ^161^ ^]^ TiO_2_,^[^ ^162^, ^163^ ^]^ NiO*_x_* ^[^ ^164^, ^165^ ^]^ and carbon electrode^[^ ^166^ ^]^ has been recently reported. Besides the significant development of the spray coating process, a maximum PCE of 19.4% has been obtained by a fully spray‐coated PSC^[^ ^167^ ^]^ (Figure 5c), indicating further research efforts are needed.

### 3.4 Flexographic Printing

**Figure 6:**
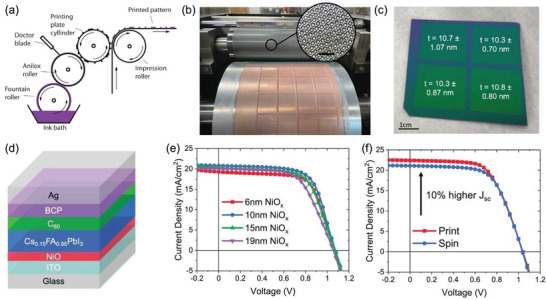
a) Schematic of flexographic printing technique. Reproduced with permission.^[^ ^175^ ^]^ Copyright 2012, Elsevier. b) Photographic image of flexographic printer printing on large substrates and the inset displaying a microscopic image of the engraved cell on the anilox roller. c) NiO*_x_* thin film flexographic printed on SiO_2_ coated Si wafer indicating thickness uniformity over a large area. d) Schematics of the device architecture, e) PCE of the PSCs with a varied film thickness of the printed NiO*_x_*, and f) comparison of *J–V* curves for PSCs fabricated by employing flexographic printed spin‐coated NiO*_x_* HTLs. Reproduced with permission.^[^ ^179^ ^]^ Copyright 2022, Wiley‐VCH.

Flexographic printing technique is a common roll‐to‐roll manufacturing method in the printing industry. As shown in **Figure** **6**a, the flexographic printing technique consists of fountain rollers that allow a steady ink transfer to the ceramic anilox roller. This roller consists of microshaped cavities embedded into its outer part, allowing ink collection, which is further transferred to the relief on a printing plate cylinder and ultimately transfers the ink to the substrate.^[^ ^168^ ^]^ The doctor blade can remove the excess ink on the anilox roller to ensure the uniform thickness of the printed layer. Flexographic printing techniques have been widely used to print the transparent electrodes and interfacial layer to fabricate polymer and organic solar cells.^[^ ^169^, ^170^, ^171^, ^172^, ^173^ ^]^ The potential of flexographic printing is that it can print with a high speed of 25 m min^‐1^, which has proven best for preparing front grid Ag electrodes for polymer solar cells.^[^ ^174^ ^]^ However, the fastest speed of the flexographic printing resulted in the irregular topology of the Ag electrode (Figure 6b), which consequently led to shunts in the solar cells and reduced the device (area of 6 cm^2^) performance to less than 2% (Figure 6c). Roll‐to‐roll flexographic printing is a relatively new technique to fabricate organic solar cells. A very limited range of functional inks has been developed, such as silver nanoparticle (Ag NP) ink or PEDOT:PSS.^[^ ^175^ ^]^

So far, flexographic printing can be of special interest for the scalable roll‐to‐roll fabrication of PSCs modules. However, an efficient active layer through flexographic printing is still one of the biggest challenges due to its complexity, as very few reports have been reported to date.^[^ ^176^, ^177^, ^178^ ^]^ In addition, the ink for flexographic printing requires polymeric binders to tune the rheological properties. However, this polymeric binder may affect the device performance and can be removed by high‐temperature annealing over 400 °C, which is not suitable for perovskite films. Because of these disadvantages, only a single report on the application of flexographic printing in PSCs has been recently reported. Huddy et al. developed ultrathin NiO*_x_* HTL by combining high‐speed (60 m min^‐1^) flexographic printing and the fastest annealing of the printed ink to fabricate PSCs.^[^ ^179^ ^]^ Also, they engineered the viscosity of the ink by varying the ratio of the solvents (2‐methoxy ethanol to ethanol) and concentration of the solute to achieve printed NiO*_x_* films with a thickness of 5–20 nm over a large area of 140 cm^2^ (Figure 6b) and high uniformity (Figure 6c). In contrast, the thickness of the spin‐coated film was 30 nm. By using these flexographic printed ultrathin NiO*_x_* HTL films, they fabricated inverted planar PSCs (Figure 6d) with device structure of glass/ITO/NiO*_x_*/Cs_0.15_FA_0.85_PbI_3_/C_60_/BCP/Ag, and achieved a PCE of 15.3% for the thickness of 10 nm (Figure 6e). The devices with printed NiO*_x_* HTLs also delivered 10% higher *J*_sc_ than the spin‐coated devices (Figure 6f). Thus, this high‐speed flexographic printing process owes a high potential for the high‐speed fabrication of both single junction and tandem devices on large‐scale flexible and rigid substrates.

### 3.5 Gravure Printing

**Figure 7:**
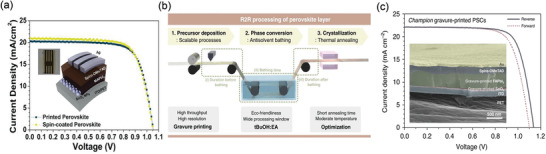
a) *J*–*V* curves of gravure printed flexible perovskite solar cells with schematic illustrations (inset). Reproduced under the Creative Common CC By license.^[^ ^86^ ^]^ Copyright 2019, The Authors, published by Wiley‐VCH. b) Schematic diagram of roll‐to‐roll processing of perovskite layers by gravure printing and c) *J*–*V* curves of gravure printed flexible perovskite solar cell with its cross‐sectional SEM image(inset). Reproduced under the Creative Common Attribution 4.0 International License.^[^ ^87^ ^]^ Copyright 2020, The Authors, published by Nature.

Gravure printing is also known as intaglio printing, which is the reverse of flexographic printing. Gravure printing technique consists of a printing cylinder with engraved patterns partially immersed in an ink container. The engraved patterns of the printing cylinder define the active area of the ink transfer. The ink is transferred from the cylinder to the substrate by applying pressure with the impression roller during the printing process. Also, a doctor blade is used to remove the excess ink from the inactive area of the printing cylinder. The print speed using a gravure printer can be beyond 18 m min^‐1^. The shape and thickness of the printed pattern depend on the pattern and the depth of the cavities in the gravure cylinder. Careful optimization of ink is required because the ink rheology, web speed, and the pressure of the impression cylinder decide the final quality of the printed layer. Kopola et al. reported organic solar cell modules with gravure printed techniques using PEDOT:PSS and P3HT:PCBM as active layer materials and obtained 1.68% power conversion efficiency with 15.45 cm^2^ area.^[^ ^180^ ^]^ Previously the same group has reported the fabrication of LED and polymer solar cells using gravure printing techniques.^[^ ^181^, ^182^ ^]^ Hu et al. first reported the use of gravure printing to produce highly oriented and large‐area perovskite nanowires for photodetectors fabrication.^[^ ^183^ ^]^ Tong et al. also reported the fabrication of cesium doped triple cation perovskite photodetector on flexible substrates using the gravure printing method under an ambient condition. It showed a broadband wavelength photoresponse in the range from UV to NIR, a high photoresponse (*R*) value of 1.62 A W^−1^, a good detectivity (D*) value up to 7.77 × 10^12^ Jones, and an excellent *I*_light_/*I*_dark_ ratio as high as 1.83 × 10^4^. In addition, these devices exhibited excellent ambient stability with high mechanical flexibility.^[^ ^184^ ^]^ In the case of PSCs, Kim et al. reported the fabrication of all printed flexible PSCs using gravure printing for the first time.^[^ ^86^ ^]^ To achieve uniform deposition of each layer, they optimized the printing inks and the processing parameters and compared the morphology, crystallography, and photovoltaic performance to the spin‐coating coating method. As a result, all‐gravure fabricated flexible PSCs showed a great PCE of 17.2%, as shown in **Figure** **7**a. The study indicated that a large area of PSCs could be developed using gravure printing as it has unique advantages such as high throughput and the ability to pattern with high resolution. In one of the recent studies, Kim et al. reported the fabrication of roll‐to‐roll flexible FAPbI_3_‐based PSCs at a pilot scale through gravure‐printing, as shown in Figure 7b.^[^ ^87^ ^]^ Also, they introduced Tert‐butanol (tBuOH) as an ecofriendly antisolvent bathing and subsequent annealing process to improve the crystallinity and uniformity of the perovskite film. As a result, they achieved a record PCE of 19.1% (Figure 7c) for the gravure printed flexible PSCs. The same group also achieved over 16% and 13% through a 100‐meter‐long roll‐to‐roll gravure printing process for SnO_2_/FAPbI_3_ and HTLs, respectively. Although a record PCE has been achieved for large‐scale PSCs by Gravure printing, it still has some disadvantages such as the limited resolution of the printing plates, high cost, and time‐consuming.

### 3.6 Screen Printing

**Figure 8:**
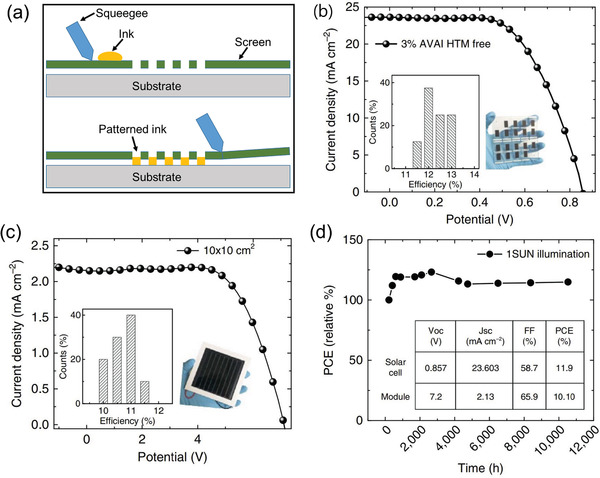
a) Schematic illustration of the screen‐printing technique. b) *J*–*V* curves of 2D/3D perovskite with 3%AVAI in HTM‐free screen‐printed PSC (FTO glass/TiO_2_/ ZrO_2_/ perovskite/ carbon), c) *J*–*V* curve using the 2D/3D perovskite with 3%AVAI in an HTM‐free 10 × 10 cm^2^ module and d) typical module stability test at the temperature of 55°. Reproduced under the Creative Commons Attribution 4.0 International License.^[^ ^195^ ^]^ Copyright 2017, The Authors, published by Nature.

The screen‐printing method is used to print the desired pattern with the help of a screen made up of thread or steel mesh which carries the printing image. This method is suitable for pastes or highly viscous ink, contrasting with the flexographic and Gravure printing methods. In the screen‐printing process, a squeegee forces the ink to pass through mesh and print on the substrate, as shown in **Figure** **8**a. Due to the high viscosity of the ink, a relatively thick film (10–500 µm) with high conductivity can be obtained, especially for electrodes. There are two types of screen printing, including flatbed and rotary screen printing. The basic principle is the same for both processes, but the operation is different. Flatbed screen printing is a stepwise process, where the screen is placed very close to the top of the substrate, the squeegee is then swiped over the screen resulting in the transfer of the ink through the mesh. Then, the screen can be lifted to change or move forward to repeat the process further. Stepwise methods are unsuitable for large‐scale roll‐to‐roll processing as they are more time‐consuming. However, flatbed screen printing is a powerful tool for small laboratory systems or in a small‐ to medium‐scale roll‐to‐roll configuration. Rotary screen printing is far better than the flatbed screen printing technique in terms of printing speed, resolution, and achievable wet thickness. Rotary screen printing is more appropriate for roll‐to‐roll production than flatbed screen printing. This technique is very simple, cost‐effective, and the making of the pattern is quick; sharp images can be produced and are feasible with different substrates like plastic, glass, and textured surfaces. The printing principle of the rotary screen‐printing method is the same as flatbed printing. However, the squeegee and the ink are assembled inside the web of the screen, which is folded into a tube. The tubular screen rotates with the same speed as the substrate, and the ink continuously pushes through the mesh by the stationary squeegee to make a full print upon every rotation. The higher processing speeds over 100 m min^‐1^ of the rotary screen‐printing process is the main advantage compared to the flatbed (0–35 m min^‐1^). However, the costly and restricted inside cleaning of the tubular screen is the disadvantage of the rotary screen‐printing technique.

Screen‐printing is widely used in PSCs for the deposition of ETLs, HTLs, and electrodes. In 2009, Kojima et al. first reported the commercialized mesoporous TiO_2_ paste deposition by screen printing to fabricate PSCs.^[^ ^15^ ^]^ They achieved a 12 µm thick mesoporous ETL by screen printing and annealing, which resulted in a PCE of 3.8%. After this successful development of PSCs, several other groups successfully screen printed mesoporous TiO_2_,^[^ ^83^, ^84^, ^85^, ^185^, ^186^ ^]^Al_2_O_3_,^[^ ^187^, ^188^ ^]^ ZrO_2_,^[^ ^185^, ^186^, ^189^, ^190^ ^]^ NiO^[^ ^187^, ^188^, ^191^ ^]^ and carbon electrodes^[^ ^192^, ^193^, ^194^ ^]^ for PSCs. As the high‐quality crystallinity of the perovskite absorber depends on the quality of the scaffold of the underlying carrier transport material, high‐quality printable mesoporous metal oxide layers are of key importance for the fully printable PSCs. In 2013, Ku et al. first reported the full printed PSCs by sequential printing of 1 µm thick TiO_2_ ETL, a 1 µm thick ZrO_2_ spacer layer, and a 10 µm thick carbon electrode where the perovskite precursor was infiltrated into these mesoporous stacks.^[^ ^83^ ^]^ This novel approach allowed them to achieve a PCE of 6.6%. Using a similar approach, Anyi et al. fabricated the full‐screen printed PSCs with 5‐ammoniumvaleric acid (5‐AVA) iodide added perovskite absorber layer.^[^ ^185^ ^]^ The 5‐AVA enhanced the crystallinity of MAPbI_3_ perovskite, contact with the mesoporous TiO_2_ scaffold with better pore filling, and reduced the defect‐related recombination, which increased the carrier lifetime and photoinduced charge separation, resulting in a certified PCE of 12.8% with high stability over 1000 h was achieved in ambient air under full sunlight. With a similar concept, in 2017, Grancini et al. also fabricated all screen‐printed PSCs with a small area (0.63 cm^2^) and modules (100 cm^2^) with carbon electrodes. By optimizing the screen‐printed layers, they achieved a PCE of 14.6% for standard mesoporous PSCs, 12.9% with carbon‐based electrodes (Figure 8b), and 11.2% for 10 cm × 10 cm perovskite solar modules (Figure 8c) with exceptional stability over 10 000 h, which enables the commercialization of PSCs (Figure 8d).^[^ ^195^ ^]^ Later in 2018, Hu et al. reported the small‐area screen printed PSCs with a bifunctional conjugated organic molecule 4‐(aminomethyl) benzoic acid hydroiodide modified perovskite layer, which further increased the PCE up to 15.6%.^[^ ^196^ ^]^

**Figure 9:**
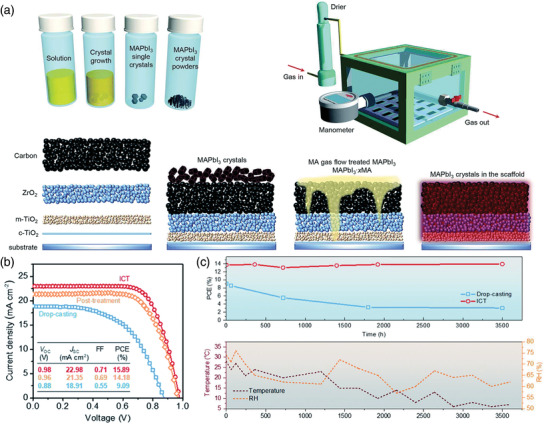
a) Schematic illustration of the: a) MAPbI_3_ single crystals and powders preparation process, ICT reactor, and ICT process from the blank scaffold to perovskites infiltrated into the ZrO_2_/m‐TiO_2_ scaffold. b) *J*–*V* curves and c) ambient and thermal stability of the screen‐printed mesoscopic PSCs (FTO glass/TiO_2_/ZrO_2_/perovskite/carbon). Reproduced under the Creative Commons Attribution‐NonCommercial 3.0 Unported Licence.^[^ ^189^ ^]^   Copyright 2020, The Authors, published by Royal Society of Chemistry.

For the commercialization of PSCs, the substrate size should be over 100 cm^2^. However, the high temperature (over 300 °C) process of the TiO_2_ based blocking and mesoporous layers causes the large substrates to crack or bend, compromising the thickness homogeneity of TiO_2_ over the substrate, affecting the performance of individual cells constituting the module. In this regard, Rossi et al. reported a low temperature processed and patterned TiO_2_ blocking layer for A4 size modules with an active area of 198 cm^2^, resulting in PCE of 6.6% after storing in the dark at the ambient condition.^[^ ^186^ ^]^ Usually, these screen‐printed mesoporous perovskite solar cells are fabricated by drop‐casting the perovskite precursor solution on the screen‐printed TiO_2_/ZrO_2_/carbon triple‐layer followed by thermal annealing. However, obtaining the complete pore filling of the perovskite into the screen‐printed mesoporous layers and high‐quality perovskite crystals is a big challenge. Therefore, Guan et al. reported the in‐situ crystal transfer (ICT) process based on gas–solid interaction to deposit single crystal MAPbI_3_ perovskite in the scaffold under methylamine gas flow. In this procedure, they first synthesized MAPbI_3_ perovskite single crystals and grounded them into powder to infiltrate into the scaffold by a homemade prototype reactor. They placed 2.5 mg of the single crystal perovskite grounded powder on the active area of the samples, which were loaded into the reactor. The perovskite powder turned into a transparent liquid state phase under the methylamine gas flow in the reactor. It spread and penetrated into the mesoporous scaffolds, which were later transformed into dark brown high‐quality MAPbI_3_ perovskite absorber when methylamine gas was replaced by N_2_ gas. This method enhanced the interconnected morphology (**Figure** **9**a) and carrier lifetime of the perovskite in the scaffold, which resulted in improved *V*_oc_ (0.98 V) and PCE of 15.89% (Figure 9b) and stability (Figure 9c).^[^ ^189^ ^]^ Apart from the screen‐printed TiO_2_ ETL‐based mesoporous PSCs, NiO has been screen printed as an HTL to improve the stability and device performance further. Cao et al. reported that inserting screen‐printed p‐type NiO between the insulating (Al_2_O_3_) layer and carbon electrode reduced the interfacial charge recombination and improved the PCE to 15.03%.^[^ ^187^ ^]^ Carbon has also been widely used as a counter electrode in the PSCs due to its hydrophobic nature, which substantially protects the perovskite layer from humidity, and its energy level aligns well with the perovskite to absorb hole carriers. Thus, it has a great potential to replace the expensive and unstable organic HTLs. Therefore, the screen printing method has been used to print carbon electrodes on both hybrid and inorganic PSCs.^[^ ^197^, ^198^, ^199^ ^]^ Recently, the screen‐printing process was used to print the front metal grid of a two‐terminal perovskite–silicon tandem solar cell.^[^ ^200^ ^]^ This method gave rise to a new low temperature processed (140 °C) silver paste prototype to fabricate tandem perovskite/c‐Si solar cells, which reduced the silver bulk resistivity, and improved the PCE to 22.6% with an aperture area of 57.4 cm^2^.

### 3.7 Inkjet Printing

**Figure 10:**
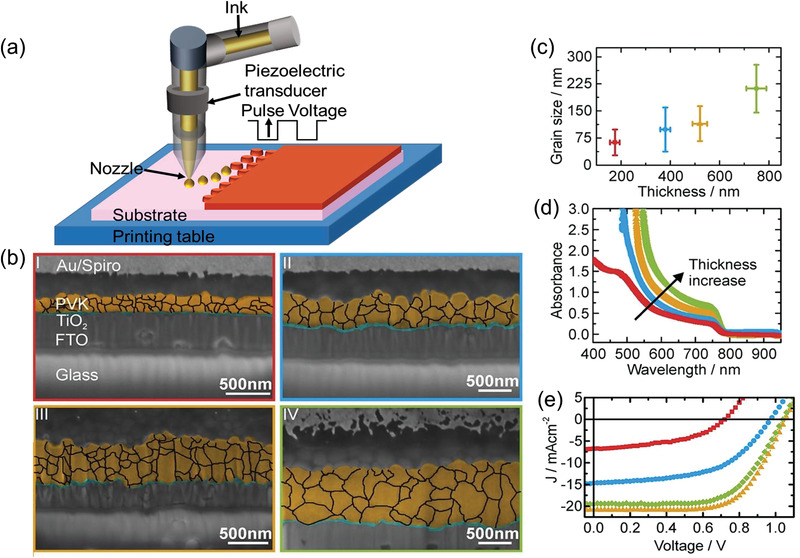
a) Schematic illustration of the inkjet printing technique. b) SEM cross‐sectional images, c) calculated grain sizes as a function of perovskite layer thickness, d) thikness dependent absorption spectra of the printed perovskite films, and e) *J–V* characteristic curve of inkjet printed triple cation PSCs (FTO glass/TiO_2_/perovskite/spiro‐OMeTAD/Au). Reproduced with permission.^[^ ^89^ ^]^ Copyright 2018, American Chemical Society.

Even though screen printing is a viable method for ambient processing of PSCs in both laboratory scale cells and roll‐to‐roll produced large area cells/modules, poor thickness control due to the viscous ink, wastage of large quantity of inks and a limited sintering time causes defects induced by the interactions of solvents from screen printing ink which inhibits the photovoltaic properties. As an alternative, inkjet printing is a feasible method to obtain desired thickness of different layers of solar cells with low cost, easily changeable digital print patterns and low material consumption, maskless and contact‐free process. The most common inkjet printing methods are drop‐on‐demand (DoD) and continuous inkjet printing. The general principle of a DoD process is that the printer head with a nozzle is mounted over the substrate table where at least one of them is movable. The printed head is connected with an ink‐filled cartridge, as shown in **Figure** **10**a. The acoustic pulse generated by either local heating of the ink or mechanical force on the ink cartridge by a piezoelectric transducer controls ink ejection from the nozzle.^[^ ^88^ ^]^ A variety of ink can be used, but the ink formulation should have appropriate conditions such as viscosity, density, and surface tension to function correctly at the nozzle of the print head.^[^ ^201^, ^202^ ^]^ The advantages of inkjet printing are that it is a reasonably high‐speed printing process, and there is virtually no wastage of materials with no production of any byproducts. The initial cost of the process is also relatively low. This method also allows for the scalability of large‐area manufacturing.^[^ ^203^ ^]^ Also, it can directly deposit versatile thin films without many intermediaries such as drums or cylinders and doctor blades. However, there are a few disadvantages of the technique, such as the print heads used in this technique are less durable and prone to clogging by the drying of the ink. Also, it is less suited for high‐volume printing. In the following paragraphs, we discuss a few attributes of the ink‐jet printing process.

**Figure 11:**
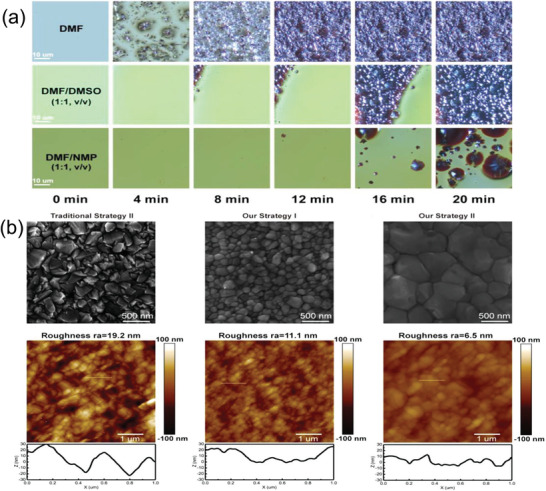
a) Images of optical microscopy for the dynamic crystallization process of the screen‐printed ink. b) Top‐view SEM and AFM images of the inkjet‐printed perovskite film using different ink systems. Reproduced with permission.^[^ ^209^ ^]^ Copyright 2020, American Chemical Society.

Inkjet printing is widely used to fabricate efficient organic and polymer photovoltaics. After discovering PSCs, the inkjet printing process was first utilized to fabricate HTL free and metal‐electrode‐free planar PSCs where nanocarbon and MAI mixed solution was inkjet printed on the spin‐coated PbI_2_ layer to in situ form MAPbI_3_.^[^ ^204^ ^]^ By this method, an interpenetrating interface was formed between MAPbI_3_ and the carbon electrode, which reduced the charge recombination at the interface and significantly improved the PCE to 11.6% (compared to the pristine device PCE of 8.5%). Later in the same year, the first one‐step solution‐processed inkjet printing of the perovskite layer was reported where the perovskite ink was prepared by dissolving PbI_2_ and MAI in *γ*‐butyrolactone (GBL).^[^ ^205^ ^]^ The perovskite precursor was inkjet printed on the top of a mesoporous TiO_2_ ETL, which resulted in a PCE of 7.9%. The PCE was further improved to 12.3% by adding an optimal concentration of MACl into the perovskite precursor and heating the substrate during inkjet printing. Similarly, the vacuum annealing step was introduced to improve the crystallinity of the inkjet printed perovskite for the fabrication of TiO_2_‐based planar PSCs, which improved the *V*_oc_ (1.0 V) and PCE (11.3%).^[^ ^206^ ^]^ Bag et al. demonstrated that multichannel inkjet printing could be helpful for in situ mixing of different ratios of cationic inks for a number of printed PSCs. They inkjet printed different ratios of MAI and FAI, with a print head connected with four ink reservoirs, on a spin‐coated PbI_2_ layer and achieved a PCE of 11.1%.^[^ ^207^ ^]^ Epoxy has been used as a protective layer to improve the stability of the inkjet printed PSCs.^[^ ^208^ ^]^ However, the thermal and moisture stability of the printed PSCs can be enhanced by modifying the perovskite absorber as well as the carrier transport layer. In this regard, inkjet printed MAPbI_3_ PSCs with PCE of 9.5% was reported to be stable more than 1000 h of continuous illumination.^[^ ^197^ ^]^ The stability was further improved with the multication approach where the perovskite precursor was prepared with a combination of triple cations such as MA‐, FA‐, and Cs‐.^[^ ^89^ ^]^ The triple cation perovskite was inkjet printed on a spin‐coated TiO_2_ ETL by optimizing the drop spacing, which influenced the grain size and thickness of the perovskite layer (Figure 10b–d). The devices delivered a high PCE of 15.3% with a high *V*_oc_ of 1.06 V, and the power output of the devices was >90% of its initial PCE over > 100 min (Figure 10e). In the inkjet printing method, the ink system plays a critical role as the ink crystallizes rapidly during printing and produces discontinuous perovskite films, which increases the defects and reduces the device performance. In this manner, a high‐throughput inkjet printing approach was reported by Chet et al., where they prepared the perovskite inks by varying the compositions (FAPbI_3_, FAPbBr_3_, MAPbI_3,_ and FAPbBr_3_) and printed the perovskite films with high speed and reproducibility.^[^ ^90^ ^]^ By this approach, they obtained a PCE of 19.0% for the MA‐based devices and 15.3% for the FA_0.75_MA_0.25_ composition‐based devices. Similarly, Li et al. reported a new ink system by composing the Cs_0.05_MA_0.14_FA_0.81_PbI_2.55_Br_0.45_ perovskite‐based printing precursor with n‐methyl pyrrolidone (NMP), DMF, and PbX_2_‐DMSO (X = Br, I) complex. NMP played a crucial role in delaying the rapid crystallization of the printed perovskite film and the PbX_2_‐DMSO (X = Br, I) complex significantly improved the quality of the perovskite layer by increasing the grain size >500 nm (**Figure** **11**a,b). As a result, the PSCs delivered PCE of 19.6% and 17.9% for device areas of 0.04 and 1.01 cm^2^, respectively. Also the large‐area device retained its original efficiency of 89% when stored in air with humidity less than 20% for 1000 h.^[^ ^209^ ^]^ Most of the reported works on the preparation perovskite films using inkjet printing methods required a vacuum‐assisted thermal annealing process to enhance the crystallization of the perovskite film which is costly and a complex process. Therefore, Zhang et al. developed a heat‐assisted inkjet printing process to directly print compact and uniform crystalline perovskites on the planar PEDOT:PSS substrates under ambient condition. By formulating precursor composition and solvent system, modifying printing temperature and optimizing the printing parameters they achieved a PCE of 16.6%.^[^ ^88^ ^]^

**Figure 12:**
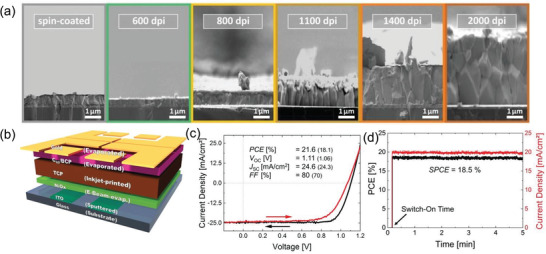
a) SEM images of cross‐sections of perovskite solar cells with inkjet perovskite absorber layers printed with different resolutions. b) Schematic illustration, c) *J–V* curves, and d) stabilized power conversion efficiency of inkjet printed PSCs. Reproduced with permission.^[^ ^30^ ^]^ Copyright 2020, Wiley‐VCH.

Along with the perovskite absorber, the carrier transport layers also play a pivotal role in improving the PCE and stability of the PSCs. In 2018, Liang et al. demonstrated a PCE of 17.0% by employing a 5 nm C60 interlayer between the TiO_2_ and inkjet printed perovskite layer.^[^ ^210^ ^]^ The C60 layer improved the wettability and significantly inhibited the heterogeneous nucleation, and improved the grain size when annealing the printed perovskite films under vacuum. Similarly, Huckaba et al. reported the inkjet printing of TiO_2_ ETLs where they prepared the ink by mixing titanium diisopropoxide bis(acetylacetonate) and TiO_2_ nanoparticle paste. With an optimal annealing temperature (400 °C), they obtained an average PCE of 15.91%.^[^ ^211^ ^]^ In addition, Buffiere et al. reported that perfect ink engineering and optimizing the inkjet printing parameters could deliver homogeneous compact TiO_2_ ETL to improve the efficiency of the printed PSCs.^[^ ^212^ ^]^ The scalable deposition of high‐quality perovskite and charge transportation layers is a crucial challenge for fabricating inkjet printed PSCs. In this regard, the first inkjet printed PSC module with an area >2 cm^2^ and PCE of 17.74% was reported in 2018.^[^ ^213^ ^]^ Later in 2018, Liang et al. demonstrated a PCE of 13.3% for a 4 cm^2^ (active area) inkjet printed PSC.^[^ ^210^ ^]^ For the scalability of the PSCs with high PCE, other emerging technologies can be combined with the inkjet printing method. Abzieher et al. reported a p–i–n structured PSC where the multication perovskite active layer was inkjet printed on the electron‐beam‐evaporated NiO*_x_* HTL and the C_60_ and bathocuproine (BCP) as ETL were deposited by the thermal evaporator. Combining these technologies with optimal conditions, they achieved a PCE as high as 20.7% with a *V*_oc_ and FF of 1.11 V and 80%, respectively. Also, they fabricated a module with an active area of 2.3 cm^2^ which delivered a PCE of 12.4 cm^2^.^[^ ^214^ ^]^ Later in 2020, the same group engineered the solvent composition of the multication perovskite precursor for inkjet printing, ink‐surface (underlying carrier transport layer) interaction and controlled the nucleation and crystallization of the deposited perovskite wet films. These optimal conditions exceptionally improved the perovskite layer thickness of >1 µm with the columnar crystal structure (**Figure** **12**a) which enabled an unprecedentedly high PCE of >21% with stabilized power output efficiency of >18% for a device architecture of ITO/NiO*_x_*/perovskite/C_60_/BCP/gold (Figure 12b–d).^[^ ^30^ ^]^ Unfortunately, the mean PCE of PSC with an active area ≈1 cm^2^ was only 10.4% due to the nonoptimized layouts. Recently, the same group reported that all inkjet‐printed p–i–n structured PSCs with a precursor‐based nickel oxide hole‐transport layer, a high‐quality inkjet‐printed triple‐cation (methylammonium, formamidinium, and cesium) perovskite absorber layer, and a double ‐layer electron‐transport layer of phenyl‐C61‐butyric acid methyl ester and bathocuproine.^[^ ^215^ ^]^ The ink properties, inkjet parameters, and the annealing procedure for each layer were optimized which delivered a PCE of >17% with low hysteresis. To prove the upscalability, they also fabricated all inkjet‐printed PSCs with a device area of 100 mm^2^ and achieved a PCE of 12.3%. In terms of electrodes, Xie et al. reported the inkjet printing of transparent silver nanowires (AgNW) as a top electrode for PSCs.^[^ ^216^ ^]^ The direct printing of AgNW on the PC_61_BM layer exhibited low device performances with low fill factor due to mismatched functions of both layers and solvent‐assisted chemical corrosion of AgNW electrode by halogen anions. Therefore, they further introduced a polyethyleneimine (PEI) thin layer in between PC61BM and AgNW electrode, which improved the device performances and stability with a higher PCE of 14.17%. Recently, Pendyala et al.^[^ ^217^ ^]^ reported the inkjet printing of transparent pillar arrays to fabricate semitransparent perovskite solar cells. The inkjet‐printed arrays of transparent pillars, composed of inert photopolymerizable liquid compositions, which are partly covered by the perovskite. The inkjet‐printed semitransparent PSC showed a PCE of 11.2% efficiency with 24% average transparency without a top metal contact.

## 4 Conclusions and Perspectives

We have reviewed the recent developments in the printed PSCs, including flexographic, gravure, screen, and inkjet printing methods. All these printing techniques have been found to be effective in producing scalable PSCs and modules. First, we discussed the blade coating technique for the PSCs, which demonstrated a PCE of over 21% for a small area (0.16 cm^2^) and over 16% with a large area of 63.7 cm^2^.^[^ ^109^ ^]^ Second, we discussed slot‐die coating, a versatile printing technique for the PSCs which delivered a PCE over 20% for a small area (0.16 cm^2^) and 15.2% for minimodules with a device area of 12 cm^2^. Third, we discussed spray‐coating, a very suitable technique for upscaling the PSCs. By optimizing the process parameters, a max PCE of 19.4% for small area (0.025 cm^2^)^[^ ^167^ ^]^ and 17.8% and 16.6% for the module sizes of 6 × 6 cm^2^ and 10 × 10 cm^2^, respectively.^[^ ^131^ ^]^ Similarly, flexographic printing can print ultrathin NiO*_x_* HTLs with a high speed of 60 m min^‐1^ over a large area of 140 cm^2^, and the PSCs fabricated with the flexographic printing technique delivered a PCE of 15.3%. However, flexographic printing requires further development of functional ink for the roll‐to‐roll fabrication of PSCs. Similarly, gravure printing has been found to be an effective technique for the roll‐to‐roll fabrication of PSCs because of its printing speed of 18 m min^‐1^, and it has unique advantages such as high throughput and the ability to pattern with high resolution. As a result, a maximum PCE of over 19% has been achieved so far by Gravure printed PSCs. However, alternative printing techniques have evolved to overcome some disadvantages such as the limited resolution of the printing plates, high cost, and time consumption of gravure printing. Screen printing can print at speeds over 100 m min^‐1^, which is faster than the flexographic and Gravure printing techniques. More importantly, screen printing has been used for printing the ETL, HTL, and the electrodes only for the screen‐printed PSCs as most reports have been reported the infiltration of the perovskite precursor into the screen‐printed mesoporous scaffold. By optimizing screen‐printed layers and improving the perovskite crystallinity by adding 5‐ammoniumvaleric acid (5‐AVA) into the perovskite precursor, improving interfacial contacts, and introducing in situ ICT process, a maximum PCE of 15.98% has been achieved. Also, the screen‐printed technique has enabled the commercialization of the PSCs as a 10 cm × 10 cm perovskite solar module has delivered a PCE of 11.2% for with an exceptional stability over 10 000. Unfortunately, the poor thickness control due to the viscous ink, wastage of large quantity of inks, and a limited sintering time limits the screen‐printing application for the commercialization of the PSCs. Therefore, inkjet printing has been found to be a feasible method to overcome these screen‐printing technique limits. Inkjet printing is used for the printing of active layer, ETL, HTL, and electrodes. However, the reports have been mainly focused on the printing of perovskite active layer by proper formulation and engineering the perovskite ink. With optimal condition a high PCE of >21% has been achieved for an inkjet‐printed PSC which is highest compared to other printing techniques such as flexographic, Gravure, and screen printing.

**Figure 13:**
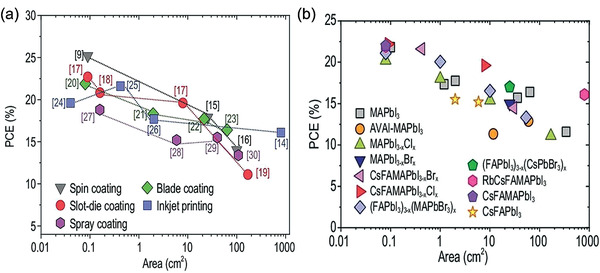
a) The champion PCEs obtained by *J*−*V* measurements with respect to the active area for different coating and printing techniques and b) with different perovskite compositions. Reproduced under the terms of the Creative Commons CC BY license^.[^ ^221^ ^]^ Copyright 2021, Chinese Academy of Science.

The above discussion indicates that all scalable printing techniques show notable progress in upscaling the PSCs. However, in terms of PCE, the printed PSCs with a small area lag behind the spin‐coated PSCs, as shown in **Table** **1**. Furthermore, the PSCs with active areas larger than 100 cm^2^ show PCEs less than 18% only, ^[^ ^158^ ^]^ whereas the devices with an active area less than 100 cm^2^ show PCEs of 20.73% (23.27 cm^2^) and 17.44% (59.33 cm^2^).^[^ ^218^ ^]^ Similarly, almost all scalable solution‐based different deposition techniques with scalable interconnection schemes for PSC modules have been demonstrated stable power output of PCE >11%, for example, slot‐die coating: 13.8% (144 cm^2^, aperture area);^[^ ^92^ ^]^ blade coating: 14.6% (57 cm^2^, aperture area);^[^ ^92^ ^]^ and spray coating: 15.5% (40 cm^2^, active area).^[^ ^219^ ^]^ In particular, from these solution‐based printing techniques for the scalability of the PSCs with high PCEs, slot‐die, and blade‐coating are the most suitable techniques to achieve high PCE over large areas. Also, the champion PCE of the PSCs and modules fabricated with different deposition techniques and perovskite compositions are shown in **Figure** **13**a,b, respectively, which indicates that the PCE gap between small‐ and large‐area devices is still enormous due to the poor control of the perovskite thin‐film homogeneity. Therefore, extensive future research and development are very essential for the commercialization of PSCs via printing techniques. The first approach should be the formulation and design of perovskite inks with optimal parameters for the printing of high quality, crack and pinhole‐free large crystal perovskite films, which can reduce the recombination induced by the intrinsic defects. Second, the compositional engineering of perovskite should be developed to improve the band alignment with the carrier transport layers and the stability of the printed PSCs. Third, effective passivation techniques should be developed which can effectively passivate the interface of the carrier transport layer/perovskite film and improve the device performance by reducing the interfacial carrier recombination. Fourth, the combination of different printing techniques for all device layer stacks, including the perovskite layer, ETL, HTL, and electrodes, is essential to reduce the PCE gaps between small area solar cells and large area modules and enable commercialization of the perovskite PV modules with active areas above 1 m^2^. Thus, these printing techniques can be very helpful in producing sheet‐to‐sheet or roll‐to‐roll perovskite modules with a cost around $0.25–$0.30 per W^[^ ^220^ ^]^

**Table 1:** Comparison of latest PCE of PSCs using spin‐coating and different printing techniques

Techniques	Area [cm^2^]	Active layer	PCE [%]	Year	Refs.
Spin coating	0.16	FAPbI_3_	25.83	2021	^[^ ^20^ ^]^
Blade coating	0.16	MAPbI_3_	21.09	2022	^[^ ^122^ ^]^
Slot‐die coating	0.16	MAPbI_3_	20.80	2021	^[^ ^150^ ^]^
Spray coating	0.025	Cs_0.05_FA_0.81_MA_0.14_PbI_2.55_Br_0.45_	19.40	2020	^[^ ^167^ ^]^
Flexographic printing	0.134	Cs_0.15_FA_0.85_PbI_3_	15.3	2022	^[^ ^179^ ^]^
Gravure printing	0.096	FAPbI_3_	19.10	2020	^[^ ^87^ ^]^
Screen printing	0.100	MAPbI_3_	15.89	2020	^[^ ^189^ ^]^
Inkjet printing	0.105	Cs_0.1_MA_0.15_FA_0.75_Pb(I_0.85_Br_0.15_)_3_	21.60	2021	^[^ ^30^ ^]^

Besides, the stability is another challenge for the commercialization of the PSCs as the longest lifetime of the PSCs has been reported to be about one year only which is significantly less than the stability of Si solar cells. Several factors are affecting the stability of the PSCs including hygroscopic organic cations vulnerable to water, oxygen, and high‐temperature; organic carrier transport materials and interfacial degradation; corrosion of metal electrodes due to light‐induced ion migration; as well as the phase segregation in the mixed halide perovskites promotes the defects migration in the perovskite layer and reduces the PCE of the PSCs. Therefore, major steps should be taken to solve these stability issues of PSCs. For example, encapsulation technology can be employed to protect the PSCs from moisture and humidity. Optimizing the chemical composition, interfacial passivation, and selection of carrier transport layers (CTLs) with high conductivity and carrier mobility can solve the ion migration and thermal instability issues of the PSCs. Furthermore, inorganic CTLs such as NiO*_x_*, CuO, CuSCN, CuI, SnO_2_, ZnO, In_2_S_3_, and MoS_2_ can be better choices over organic CTLs because of their superior moisture and thermal stability. Finally, the surface passivation technique should be developed to effectively suppress the defects on the large perovskite surface and improve the PCE and stability of the large PSC modules. Therefore, these techniques can be beneficial to obtain high‐performance and stable, large‐area PSCs in the near future.

## Conflict of Interest

The authors declare no conflict of interest.
